# Predicting the Potential Spread of Invasive Reptiles From Hong Kong and Taiwan to Other Regions of China

**DOI:** 10.1002/ece3.72947

**Published:** 2026-01-19

**Authors:** Chaosheng Mu, Jichao Wang

**Affiliations:** ^1^ Ministry of Education Key Laboratory for Ecology of Tropical Islands, Key Laboratory of Tropical Animal and Plant Ecology of Hainan Province, College of Life Sciences Hainan Normal University Haikou China

**Keywords:** climate change, invasion risk assessment, Maxent, range expansion, species distribution models, suitable habitats

## Abstract

Biological invasions pose significant threats to global ecosystems, with invasive reptiles causing particular concern due to their increasing spread through international trade and potential range expansion under climate change. This study investigated the potential spread of five invasive reptile species that have established breeding populations in Hong Kong and Taiwan but have not yet invaded mainland China. Using the Maximum Entropy algorithm in Species Distribution Models, we integrated global occurrence records with current and future environmental variables to predict suitable habitats and potential distribution changes under different climate scenarios. We assess invasion risk based on the current and future distribution ranges of suitable habitats for invasive species. Our study results indicate the brown anole ( *Anolis sagrei* ) has the widest predicted distribution range, with suitable habitats across most regions of China, thus posing the highest invasion risk. The veiled chameleon ( *Chamaeleo calyptratus* ), Brook's house gecko ( *Hemidactylus brookii* ), and the green iguana ( *Iguana iguana* ) also present elevated invasion risks, as their suitable habitats are primarily located in southern China. In contrast, the monarch gecko ( *Gekko monarchus* ) has extremely low invasion potential, with only a small number of suitable habitats found along the southern coastal regions of China. Temperature and precipitation emerged as the primary factors influencing species distribution. Future climate projections indicate that the suitable habitats for all species will significantly expand, with distribution centers notably shifting northward and inland, particularly under high greenhouse gas emission scenarios. This study underscores the importance of species‐specific management strategies and enhanced biosecurity measures, especially in regions identified as high‐risk areas. It provides valuable evidence for developing proactive measures to prevent the spread of these high‐risk invasive reptiles from Hong Kong and Taiwan into mainland China.

## Graphical abstract

This study used species distribution models that combined global occurrence records with current and future environmental variables to predict the potential distributions of five invasive reptile species that have established breeding populations in Hong Kong and Taiwan but have not yet invaded mainland China. The results show that *Anolis sagrei* poses the highest invasion risk with the widest predicted distribution, followed by *Chamaeleo calyptratus* , *Hemidactylus brookii* , and *Iguana iguana* , while *Gekko monarchus* has very limited invasion potential, with temperature and precipitation being the primary factors influencing distribution. Under future climate scenarios—especially high greenhouse gas emission pathways—suitable habitats for all species are predicted to significantly expand, with distribution centers shifting notably northward and inland, underscoring the need for species‐specific management strategies and enhanced biosecurity measures.

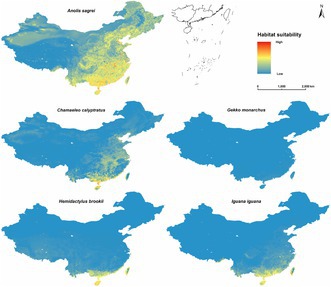

## 1 Introduction

Biological invasions, the establishment and spread of alien species in non‐native environments, are considered a major threat to global ecosystems, profoundly impacting biodiversity and ecosystem services (Ricciardi et al. 2017; Simberloff et al. 2013). Accelerated globalization has significantly increased the spread and establishment of invasive species (Seebens et al. 2017). Similarly, the incidence of reptile invasions is rising, primarily driven by the pet trade and accidental transportation through global commerce (Frost et al. 2019). This is particularly relevant in rapidly developing regions where urbanization and international trade create numerous opportunities for reptile introduction and establishment (Dawson et al. 2017; X. Liu et al. 2019). Invasive reptiles have caused substantial ecological damage, including the extinction of native species, disruption of food webs, and alteration of ecosystem processes (Kraus 2015).

Against the backdrop of climate change, the dynamics of invasive species are shifting (Bellard et al. 2013). Rising temperatures and altered precipitation patterns may create new opportunities for range expansion, particularly for ectothermic species like reptiles (Urban et al. 2016). Climate change could significantly affect the distribution of invasive reptiles by altering habitat suitability and creating new invasion corridors (Bellard et al. 2018). Furthermore, changes in human land use patterns and urbanization may interact with climate change to facilitate invasive species establishment in previously unsuitable areas (X. Liu et al. 2020).

Socioeconomic factors, particularly GDP per capita, population density, and economic activity intensity, significantly enhance the likelihood of biological invasion (Dawson et al. 2017; Floerl et al. 2009). These factors create multiple introduction pathways and facilitate species dissemination through increased trade connectivity and transportation networks (Floerl et al. 2009). Taiwan and Hong Kong, as frontiers of China's economic growth and external openness (Chow and Lin 2002; H. Li et al. 2009), have become hotspots for invasive alien species and may serve as stepping stones for these species to spread into mainland China (Lu et al. 2018). Previous studies have shown that Hong Kong and Taiwan report the highest number of newly recorded invasive alien species in China (Lu et al. 2018). Additionally, regions adjacent to these areas tend to record higher numbers of newly identified invasive alien species (Lu et al. 2018). However, whether the alien species that have already invaded Hong Kong and Taiwan will continue to spread to other regions of China remains under‐researched.

Predicting the potential spread of established invasive reptile populations is essential for developing effective management strategies (X. Liu et al. 2020). Species Distribution Models (SDMs) have emerged as valuable tools for assessing invasion risk and identifying areas susceptible to future invasion (Bellard, Leroy, et al. 2016). Species Distribution Models integrate species occurrence data with environmental variables to predict suitable habitats where invasive species might establish and spread (Peterson 2003). In this study, we focus on five invasive reptile species that were introduced from foreign regions and have established breeding populations in Hong Kong and/or Taiwan but have not yet spread to mainland China. The species include the brown anole ( *Anolis sagrei* ) (Wang 2013), the veiled chameleon ( *Chamaeleo calyptratus* ) (Lee et al. 2019), the monarch gecko ( *Gekko monarchus* ) (Lee et al. 2019), Brook's house gecko ( *Hemidactylus brookii* ) (Lee et al. 2019; Liu 2000), and the green iguana ( *Iguana iguana* ) (Lee et al. 2019; Mo and Mo 2022). We used global occurrence records of these five reptile species, along with global climate and land cover data, to simulate the potential distribution of these species in China. Our research has two objectives: (1) assess the potential distribution range of these five invasive reptile species under current environmental conditions in China; and (2) evaluate how future climate change scenarios might affect their potential distribution patterns. Our findings will provide valuable insights for developing proactive management strategies to prevent the spread of these invasive reptiles.

## 2 Materials and Methods

### 2.1 Occurrence and Environmental Data

Through literature research, we identified five species of invasive reptiles that have established breeding populations in Hong Kong and/or Taiwan, China. The species occurrence data we used was sourced from publicly available data published by Mi et al. (2023), encompassing global occurrence records of amphibians and reptiles from 1970 to 2022. The data primarily originates from online databases, field surveys, museum records, and published scientific literature, excluding records related to capitals, institutions, museums, and potential erroneous entries. Occurrence records outside the range of the global bioclimatic variables were excluded. To mitigate the impact of sampling bias, we employed the “spThin” package in R (version 4.3.0) for spatial thinning, using a minimum distance of 10 km between pairs of occurrence records (Aiello‐Lammens et al. 2015). The final numbers of occurrence records retained for each species were: *A. sagrei* (1340), *C. calyptratus* (44), *G. monarchus* (95), *H. brookii* (249), and *I. iguana* (1838). To avoid underestimating potential distribution areas by solely relying on native habitat data, we adopted a global occurrence data calibration model (Chapman et al. 2019; Mainali et al. 2015).

We sourced six global bioclimatic and elevation variables from the WorldClim database (Fick and Hijmans 2017). These bioclimatic variables represent climatic conditions that limit the survival and reproduction of reptiles, including Annual Mean Temperature (Bio1), Isothermality (Bio3), Maximum Temperature of the Warmest Month (Bio5), Minimum Temperature of the Coldest Month (Bio6), Mean Annual Precipitation (Bio12), and Precipitation During the Warmest Quarter (Bio18) (Araújo et al. 2006; X. Li et al. 2016). The current climatic variables are based on long‐term average data from 1970 to 2000. To avoid multicollinearity in the statistical models, we removed bioclimatic variables with an absolute Pearson correlation coefficient greater than 0.7 (Dormann et al. 2013). Water resources are crucial for the survival of wildlife (Rosenstock et al. 1999); therefore, we generated continuous data on the distance to water bodies using the 2015 land cover data published by Chen et al. (2022) as the current data. Vegetation cover significantly affects the distribution of reptiles; therefore, we used the 2015 percentage data of forest, non‐forest, and crop vegetation cover published by Chen et al. (2020) as the current dataset. For future predictions, we selected bioclimatic variables from three Shared Socioeconomic Pathways (SSPs): SSP126 (sustainable development), SSP245 (middle of the road development), and SSP585 (fossil fueled development) (Fan et al. 2020). To account for uncertainty in future climate projections, we selected three Global Circulation Models (GCMs): BCC‐CSM2‐MR, IPSL‐CM6A‐LR, and MIROC6, for the years 2050 (average for 2041–2060) and 2090 (average for 2081–2100). We selected future land cover data that correspond to the same time and SSPs as the bioclimatic data. Although the distance to water body data does not differentiate among different GCMs, the percentage data for the three types of vegetation cover provides information from five GCMs, including GFDL‐ESM2M, HadGEM2‐ES, IPSL‐CM5A‐LR, MIROC5, and NorESM‐M. Future environmental data are derived by calculating the average of multiple GCMs. Bioclimatic and elevation variables utilize a resolution of 2.5 min (equivalent to 4.6 km × 4.6 km). To ensure consistency with the bioclimatic variables, we resampled the distance to water bodies data (1 km × 1 km) and the vegetation cover percentage data (0.05°, equivalent to 5.5 km × 5.5 km) to the 2.5 min spatial resolution. All these future data originated from Phase 6 of the Coupled Model Intercomparison Project (CMIP6).

### 2.2 Maxent Model

The study employed Maximum Entropy modeling (Maxent) as the primary tool to predict habitat distribution and potential changes due to climate change. Maxent is a presence‐only algorithm that estimates the probability distribution of species occurrence by contrasting environmental conditions at known presence sites with those at background locations (Phillips et al. 2006). Because absence data are not used, the model reflects relative habitat suitability rather than true probability of occurrence (Elith et al. 2011). Despite this limitation, Maxent has been shown to provide high predictive accuracy and robustness, and it remains one of the most widely applied Species Distribution Models (SDMs) worldwide (Rathore and Sharma 2023). Parameter optimization in SDMs is essential, as it involves identifying the optimal combination of parameters that not only enhances predictive performance but also ensures applicability across diverse ecological scenarios and the validity of scientific research (Cobos et al. 2019; Steele and Werndl 2013). This process was facilitated by the use of the R 4.3.0 program and the kuenm package (Cobos et al. 2019), through which 31 possible combinations of feature types (linear, quadratic, product, threshold, and hinge) and 10 regularization multiplier settings (0.1, 0.3, 0.6, 0.9, 1, 2, 3, 4, 5, and 6) were evaluated. The selection of the optimal parameters was guided by three criteria: statistical significance, assessed through the partial ROC method, which tests whether model performance is significantly better than random expectations (Peterson et al. 2008); predictive power, evaluated via the omission rate (Anderson et al. 2003); and model complexity, determined by the Akaike Information Criterion corrected (AICc value) (Warren et al. 2010), with a preference for the model exhibiting the lowest AICc value (delta AICc = 0), as it is indicative of the best model (Cobos et al. 2019). The optimal feature types and regularization multiplier for the final Maxent model were determined through this optimization process. The maximum number of background points was set to 10,000. Calibration of the model utilized 70% of occurrence records, reserving the remaining 30% for validation and assessment of the model's predictive accuracy (Elith et al. 2011; Phillips et al. 2006). Clamping settings were used during extrapolation. Clamping constrains feature values in novel environments to remain within the range observed in the training data, which helps reduce uncertainties when the model is projected into environmental conditions not encountered during the training phase (Elith et al. 2011). Model robustness was enhanced by performing 10 replicate runs with cross‐validation, and the logistic output format was selected, with all other parameters set to Maxent's default values for consistency (Elith et al. 2011; Phillips et al. 2006). Finally, the results for the five species were imported into ArcGIS 10.5 (ESRI, Redlands, CA, USA) and converted into raster data using a conversion tool. The results were standardized using the geographic information system toolkit SDMtoolbox 2.5 (Brown et al. 2017), which is based on Python 2.7, before being summed. To assess the predictive accuracy of the species distribution models generated by Maxent, we used two complementary metrics: the Area Under the Receiver Operating Characteristic Curve (AUC) and the Continuous Boyce Index (CBI) (Boyce et al. 2002; Jiménez‐Valverde 2012). The AUC measures the model's ability to discriminate presence records from background points (Jiménez‐Valverde 2012), whereas the CBI evaluates the agreement between predicted habitat suitability and observed presences (Boyce et al. 2002). The CBI was calculated using the ecospat package in R (Di Cola et al. 2017). Values of both metrics closer to 1 indicate better predictive performance. We quantified the relative importance of each environmental variable in the Maxent model using the Percentage Contribution method. This method calculates the contribution of each variable based on how much it increases the model's gain during the training process, attributing increases in the regularized training gain to the environmental variables that most improve the model (Phillips et al. 2006).

### 2.3 Changes in Suitable Habitat Area

In our study, we employed the 10th percentile training presence threshold method to convert the predictive distribution maps generated by Maxent into binary maps of suitable and unsuitable habitats. This threshold is set at the value above which 90% of the training presence records are predicted, effectively excluding the lowest 10% of suitability scores associated with known occurrences (Capinha et al. 2013). To further examine trends in species invasiveness, we used SDMtoolbox and binary suitability maps to perform two analyses under future environmental change scenarios in China. Specifically, we quantified the areas and proportions of suitable habitat expansion and contraction for each species and calculated shifts in the geographic centroids of species' distributions to elucidate potential invasion dynamics over the coming decades (Brown et al. 2017).

## 3 Results

### 3.1 Model Parameter Selection and Accuracy Evaluation

We generated 310 candidate models for each species, and ultimately, only one set of model parameters per species met our screening criteria (Table 1). The mean AUC and CBI values for ten replicate runs of each species were as follows: *A. sagrei* (AUC = 0.823, CBI = 0.896), *C. calyptratus* (AUC = 0.979, CBI = 0.873), *G. monarchus* (AUC = 0.985, CBI = 0.914), *H. brookii* (AUC = 0.950, CBI = 0.990), and *I. iguana* (AUC = 0.869, CBI = 0.911). Both AUC and CBI results demonstrated strong model performance for all species, with AUC values exceeding 0.8 and CBI values above 0.85, indicating reliable discrimination between suitable and unsuitable habitats. Among them, *C. calyptratus* , *G. monarchus* , and *H. brookii* exhibited the highest predictive accuracy, with AUC values above 0.95 and CBI values approaching 0.9 or higher, reflecting highly consistent habitat suitability predictions (Boyce et al. 2002; Elith et al. 2011; Phillips et al. 2006).

**TABLE 1:** Performance of the Maxent model under optimal parameters for each species.

Species	Features	RM	Mean_AUC_ratio	*p*val_pROC	5% omission rate	AICc	delta_AICc	W_AICc
*A. sagrei*	q	6.0	1.30	0	0.04	22,482.65	0	1.00
*C. calyptratus*	lpt	3.0	1.82	0	0.00	565.61	0	0.77
*G. monarchus*	lqt	4.0	1.92	0	0.03	1132.63	0	0.99
*H. brookii*	lqt	3.0	1.72	0	0.04	3598.91	0	1
*I. iguana*	lpt	0.1	1.54	0	0.05	29,504.04	0	1.00

Abbreviations: 5% omission rate, percentage of test presences incorrectly predicted as unsuitable; AICc, corrected Akaike information criterion; delta AICc, difference from the best model; mean_AUC_ratio, ratio of mean AUC to random expectation; *p*val_pROC, partial ROC test *p* value; RM, regularization multiplier; W_AICc, AICc weight.

### 3.2 Predict the Current Distribution of Invasive Species

**FIGURE 1:**
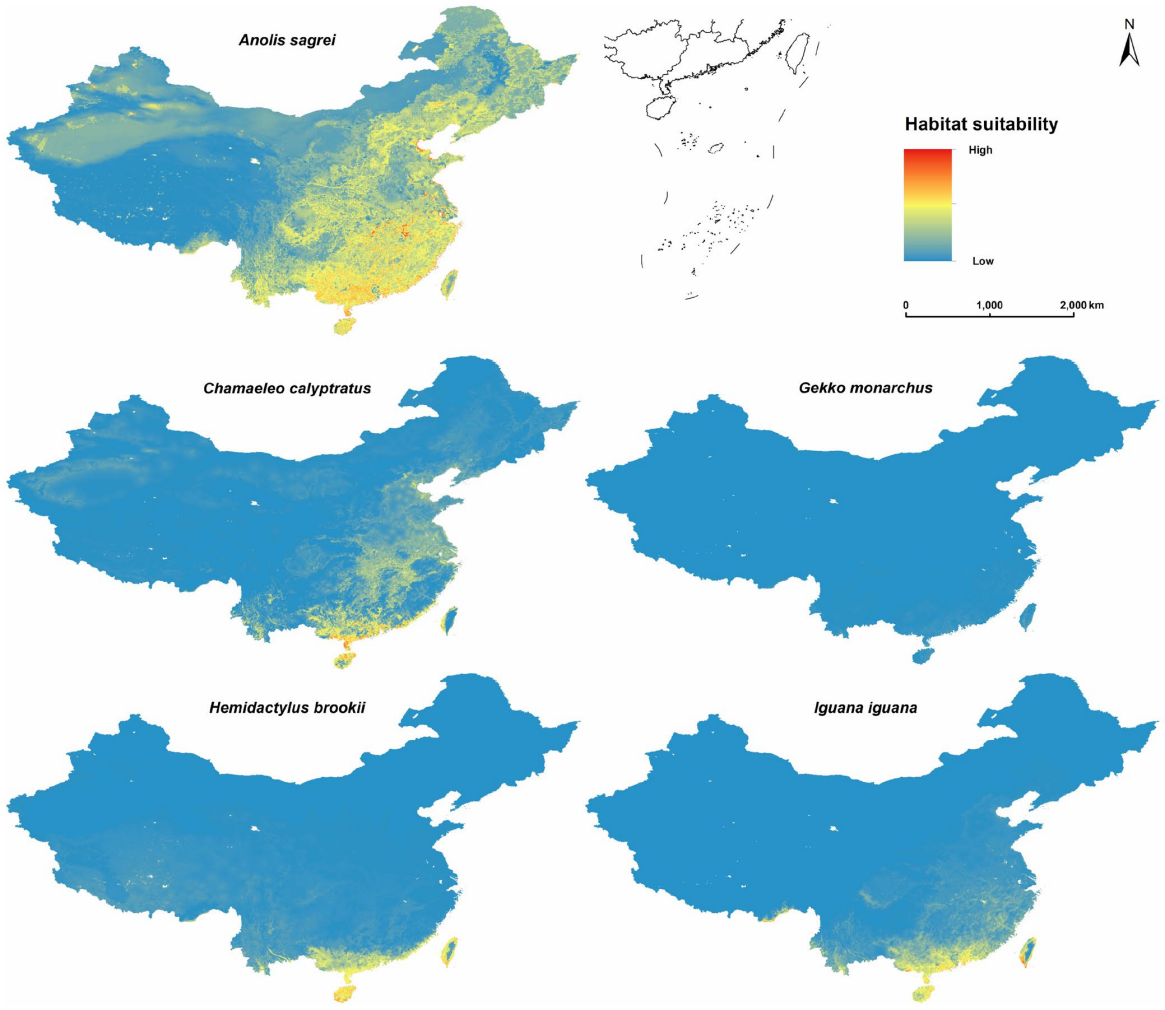
Habitat suitability prediction maps for five invasive reptiles in China under current climate and land cover conditions.

Although the suitable habitats of the five invasive reptile species differ markedly in both range and intensity, all of them show high habitat suitability in southern China, particularly in the coastal regions (Figure 1). *A. sagrei* displayed a potentially widespread distribution range in China, with high habitat suitability values across southeastern China and moderate suitability extending into central regions. The areas of highest predicted suitability were concentrated in southern China and the southern portions of eastern China. *C. calyptratus* showed a more restricted distribution pattern, with areas of high habitat suitability primarily in southern China, while moderate suitability areas extended into parts of central China. The potential distribution range of *G. monarchus* in China is very narrow, with only low‐suitability habitats located in the southern coastal regions. *H. brookii* and *I. iguana* showed similar distribution patterns, with suitable habitats concentrated in southern China. Both species' highest habitat suitability areas were predicted in the coastal areas of southern China, with moderate suitability areas extending slightly northward along the southeastern coast (Figure 1).

The results indicate that although different species exhibit varying sensitivities to environmental variables, temperature and precipitation are overall the primary factors influencing their distribution (Table 2). Annual Mean Temperature contributes notably to the models of *I. iguana* and *A. sagrei* , accounting for 40.1% and 31.30% of their model contributions, respectively. Additionally, Isothermality and Minimum Temperature of the Coldest Month are also key factors. Specifically, Isothermality contributes notably to the models of *C. calyptratus* , *G. monarchus* , and *I. iguana* , accounting for 30.4%, 31.7%, and 38.9% of their model contributions, respectively. Minimum Temperature of the Coldest Month has the greatest contribution to *H. brookii* , accounting for 69.3%. Mean Annual Precipitation plays an important role for *G. monarchus* , accounting for 42.5%. Furthermore, Non‐Forest Cover Percentage has a significant impact on the distribution of *A. sagrei* , accounting for 31.2% of its model contribution. Altitude plays an important role in the model for *C. calyptratus* , accounting for 25.1% (Table 2).

**TABLE 2:** Average percentage contribution of environmental variables for each species in the Maxent model.

Species	Bio_1	Bio_3	Bio_5	Bio_6	Bio_12	Bio_18	Altitude	Distance	Forest	Non‐forest	Crop
*A. sagrei*	31.3	×	9.1	×	0	0	0.2	0.5	12.7	31.2	15.0
*C. calyptratus*	21.3	30.4	×	×	2.2	×	25.1	6.6	3.7	4.6	6.1
*G. monarchus*	3.2	31.7	×	×	42.5	2.1	0	2.3	7.8	6.2	4.0
*H. brookii*	3.0	1.8	×	69.3	4.4	3.8	0.6	5.7	4.7	0.5	6.4
*I. iguana*	40.1	38.9	1.8	×	1.9	2.1	1.5	6.4	1.6	4.8	1.1

*Note:* “×” indicates that the variable was not used in the model for that species.

Abbreviations: Bio 1, Annual Mean Temperature; Bio 12, Annual Precipitation; Bio 18, Precipitation of Warmest Quarter; Bio 3, Isothermality; Bio 5, Maximum Temperature of the Warmest Month; Bio 6, Minimum Temperature of the Coldest Month; Crop, land‐cover types; Distance, distance to water bodies; Forest, non‐forest.

### 3.3 Future Changes in the Distribution of Invasive Species

**FIGURE 2:**
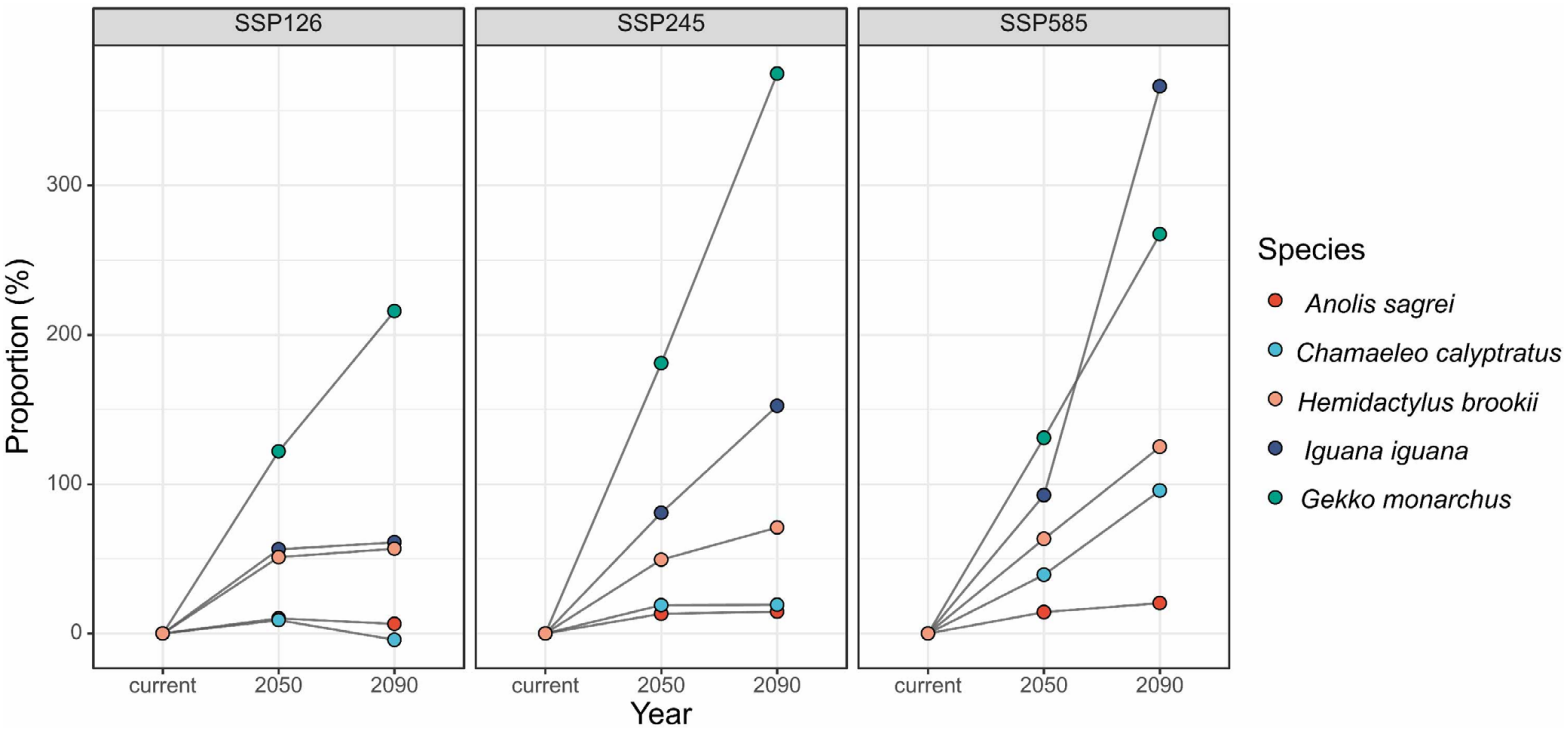
Proportion of suitable habitat growth for five invasive reptiles in China in 2050 and 2090 under different Shared Socioeconomic Pathways (SSPs). The SSPs include SSP126 (low‐emission), SSP245 (moderate‐emission), and SSP585 (high‐emission) scenarios.

**FIGURE 3:**
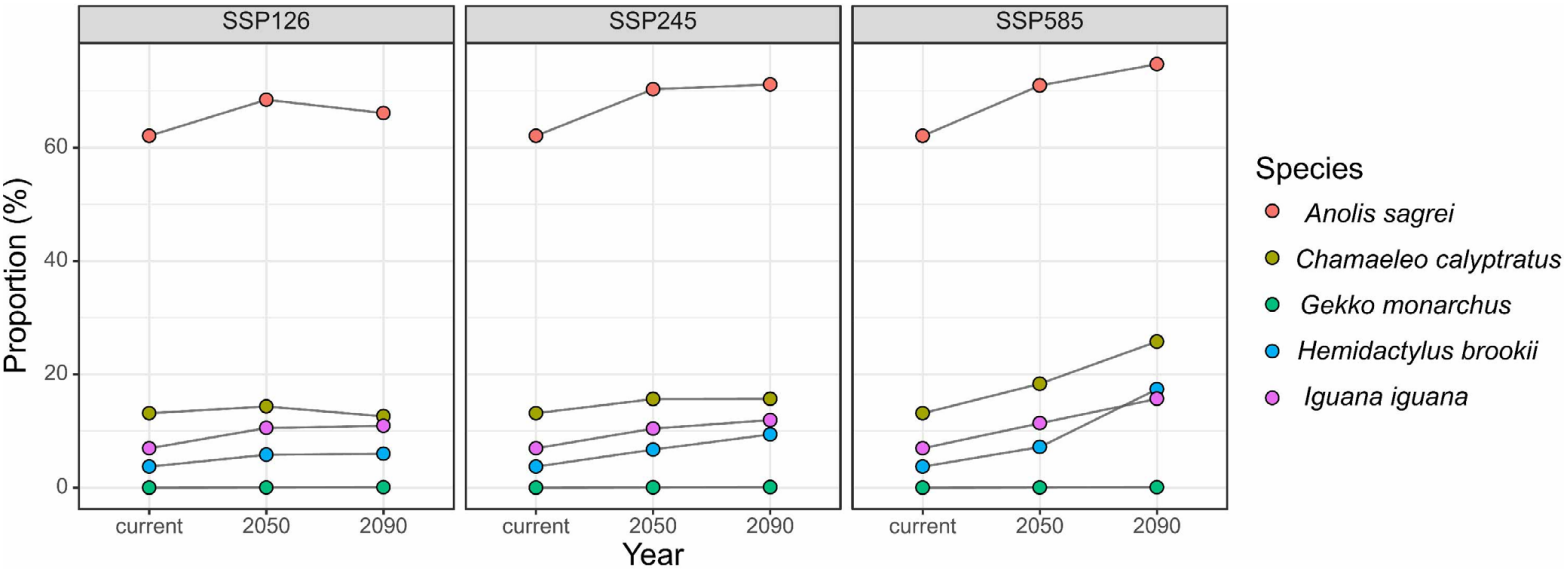
Changes in the proportion of suitable habitat for five invasive reptiles in China in 2050 and 2090 under different Shared Socioeconomic Pathways (SSPs). The SSPs include SSP126 (low‐emission), SSP245 (moderate‐emission), and SSP585 (high‐emission) scenarios.

**FIGURE A1:**
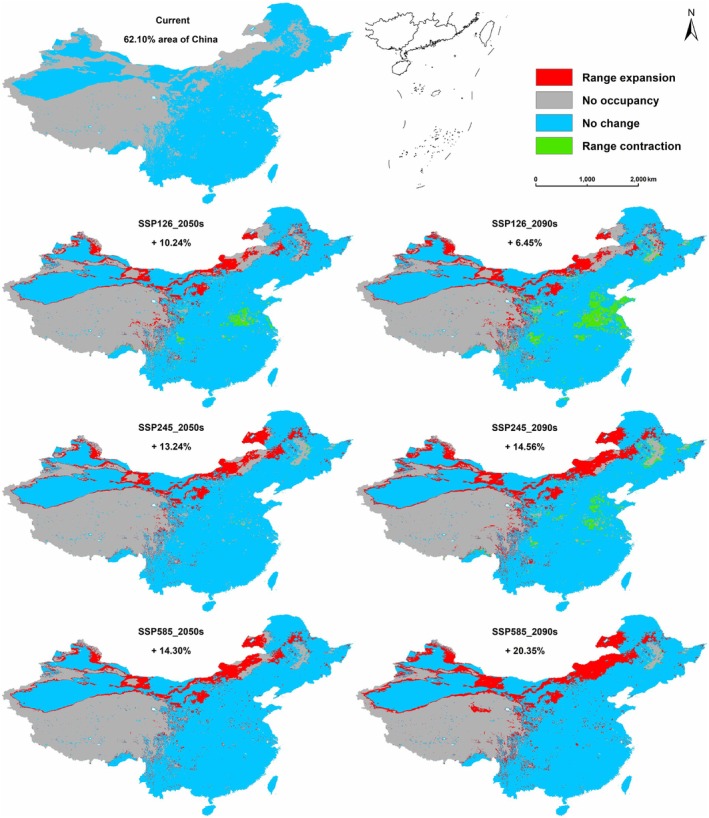
Expansion and contraction of the activity ranges of *Anolis sagrei* in China in 2050 and 2090 under different Shared Socioeconomic Pathways (SSPs). The SSPs include SSP126 (low‐emission), SSP245 (moderate‐emission), and SSP585 (high‐emission) scenarios.

**FIGURE A2:**
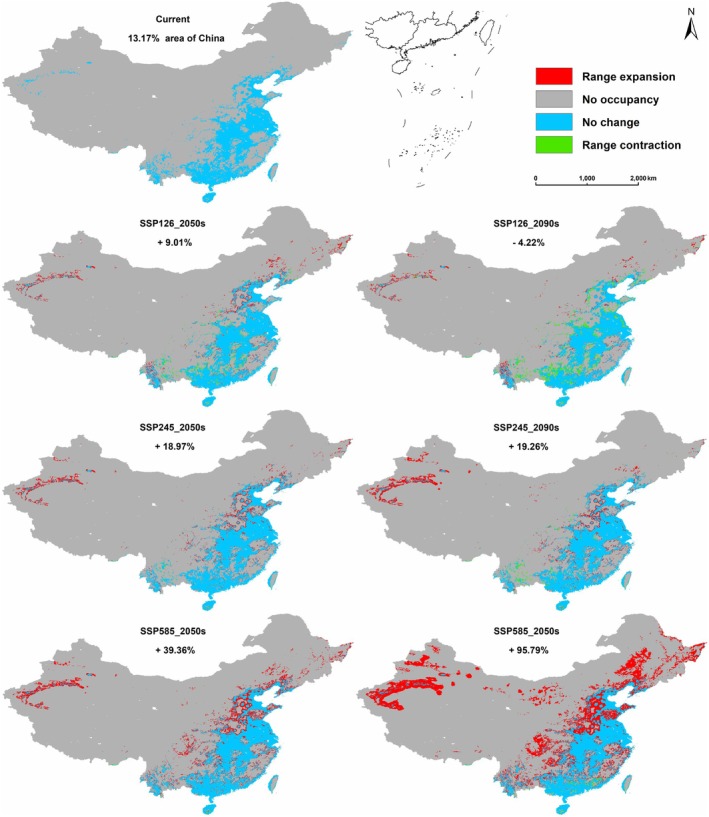
Expansion and contraction of the activity ranges of *Chamaeleo calyptratus* in China in 2050 and 2090 under different Shared Socioeconomic Pathways (SSPs). The SSPs include SSP126 (low‐emission), SSP245 (moderate‐emission), and SSP585 (high‐emission) scenarios.

**FIGURE A3:**
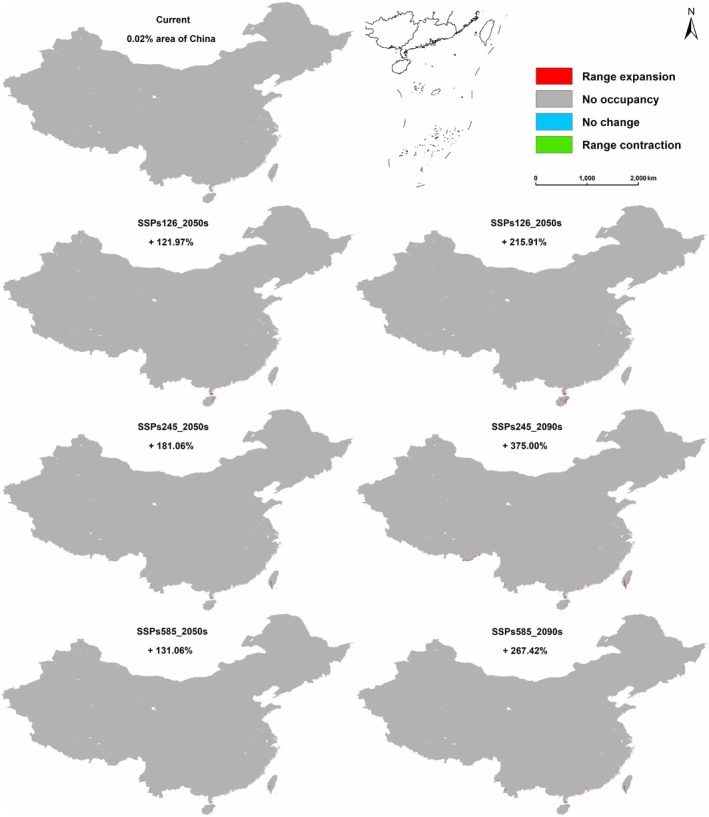
Expansion and contraction of the activity ranges of *Gekko monarchus* in China in 2050 and 2090 under different Shared Socioeconomic Pathways (SSPs). The SSPs include SSP126 (low‐emission), SSP245 (moderate‐emission), and SSP585 (high‐emission) scenarios.

**FIGURE A4:**
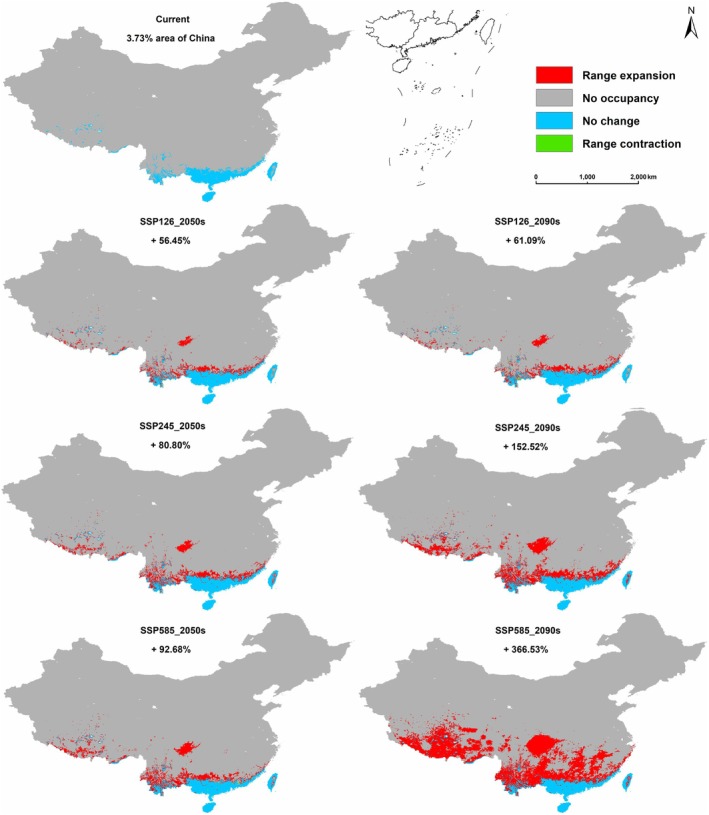
Expansion and contraction of the activity ranges of *Hemidactylus brookii* in China in 2050 and 2090 under different Shared Socioeconomic Pathways (SSPs). The SSPs include SSP126 (low‐emission), SSP245 (moderate‐emission), and SSP585 (high‐emission) scenarios.

**FIGURE A5:**
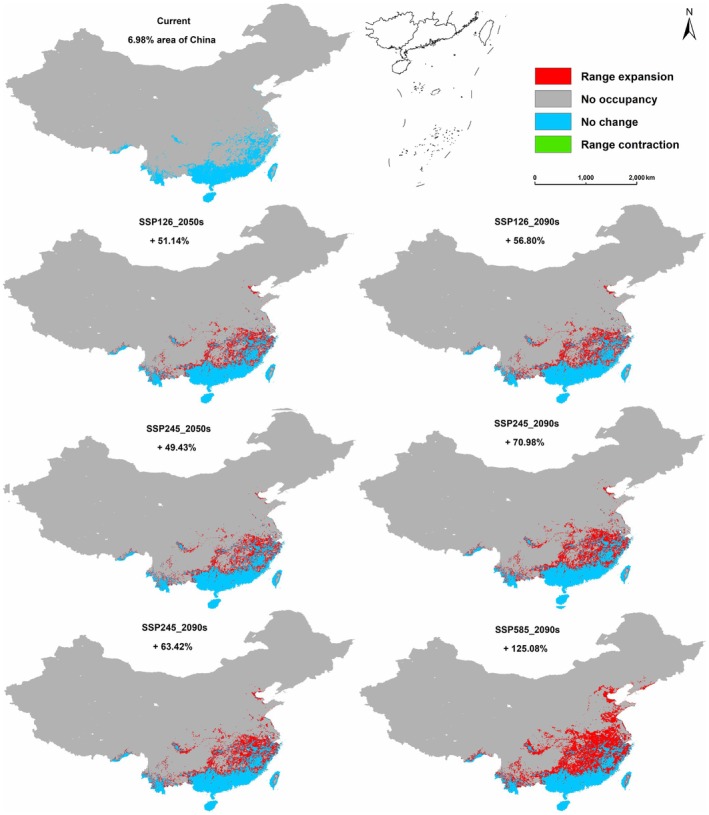
Expansion and contraction of the activity ranges of *Iguana iguana* in China in 2050 and 2090 under different Shared Socioeconomic Pathways (SSPs). The SSPs include SSP126 (low‐emission), SSP245 (moderate‐emission), and SSP585 (high‐emission) scenarios.

The 10th percentile training presence threshold values were 0.097 for *A. sagrei* , 0.095 for *C. calyptratus* , 0.089 for *G. monarchus* , 0.092 for *H. brookii* , and 0.086 for *I. iguana* . Under future climate and land cover changes, the suitable habitats of these five invasive reptile species are projected to expand notably. The expansion patterns vary among different climate scenarios, with SSP585 showing the most significant expansion potential (Figure 2). *Anolis sagrei* , which currently has the largest distribution range (62.10% of China's area), shows moderate expansion ranging from 6.45% to 20.35% (Figure A1). *C. calyptratus* exhibited a different response, expanding by 95.79% under the SSP585 scenario in 2090, but showing a slight contraction of 4.22% under the SSP126 scenario (Figure A2). Despite the limited distribution range of *G. monarchus* , it demonstrates significant expansion potential, with a 375% increase under the SSP245 scenario in 2090 (Figure A3). The distribution range of suitable habitats for *H. brookii* has also notably expanded, with a 366.53% increase under the SSP585 scenario in 2090 (Figure A4). The distribution of *I. iguana* is projected to expand by 49.43% to 125.08% in the future (Figure A5). In terms of the overall distribution range in the future, *A. sagrei* remains the species with the widest coverage of suitable habitats. The distribution ranges of the other four species' suitable habitats in 2050 will not exceed 20%. By 2090, only *C. calyptratus* under the SSP585 scenario will reach a distribution range of 20%–30%, while others will remain below 20%. Notably, *G. monarchus* consistently maintains an extremely low proportion of total area despite its high expansion rate (Figure 3).

**FIGURE 4:**
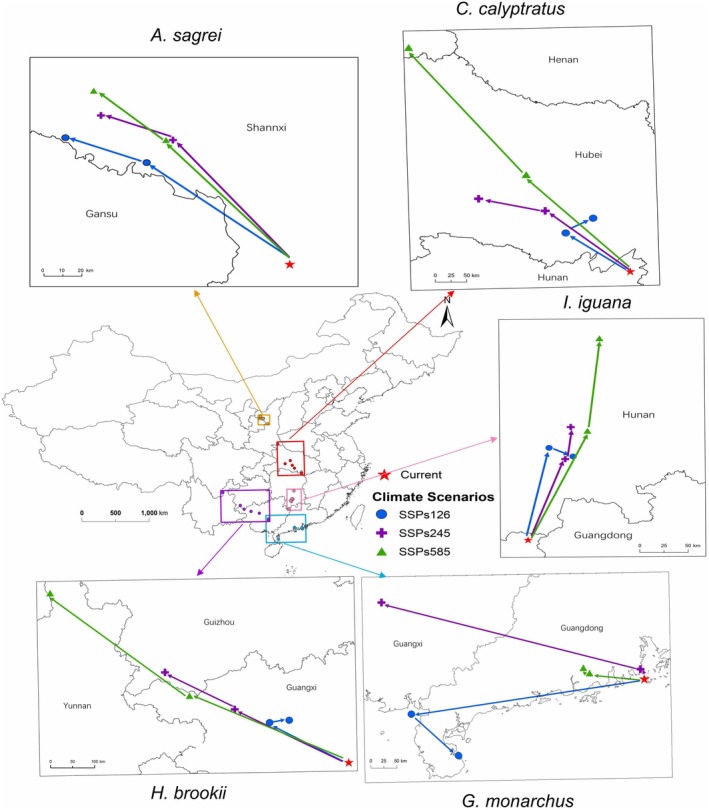
Shift in suitable habitat centroids for five invasive reptiles in China under different Shared Socioeconomic Pathways in 2050 and 2090 (SSPs). The SSPs include SSP126 (low‐emission), SSP245 (moderate‐emission), and SSP585 (high‐emission) scenarios.

In the future, the distribution centers of various invasive species are projected to experience geographical shifts under changing climate conditions. *Anolis sagrei* , *C. calyptratus* , and *H. brookii* are expected to shift northwestward, *G. monarchus* westward, and *I. iguana* northward. Overall, the centroid positions of all species show a general northward and inland shift pattern (Figure 4).

## 4 Discussion

Our research is the first to use SDMs to investigate the potential for invasive species to further invade other regions of China after entering Hong Kong and Taiwan. Previous studies have primarily focused on the spread of invasive species that have already established stable breeding populations in inland China (Gong et al. 2023; Mu et al. 2022; Peng et al. 2020). However, research evaluating the potential invasion risks of reptile species that have established populations in economically developed regions of China remains quite limited. Additionally, the effects of future climate change on the invasion potential of these species have not been adequately studied.

Our models reveal important patterns in the invasion risk of the five reptile species across China under current environmental conditions. *Anolis sagrei* emerges as the species posing the highest invasion risk, with suitable habitats extending across much of southern and eastern China. The broad environmental tolerance of *A. sagrei* , particularly its ability to thrive in human‐modified landscapes, further increases its invasion potential (Battles et al. 2018; Stroud et al. 2017). In contrast, the invasion risk of *G. monarchus* is extremely low, as its suitable habitat is limited to a few coastal areas in the southern regions, indicating that this species may not require immediate attention for widespread invasion concerns. *Chamaeleo calyptratus* shows particularly concerning invasion potential in tropical and subtropical regions of China, likely due to its strong adaptability to warm climates and arboreal habitats (Krysko et al. 2004). The potential distribution of *H. brookii* and *I. iguana* is primarily concentrated in southern China. Having established invasive populations across Southeast Asia, *H. brookii* has successfully colonized buildings and other artificial structures in urban environments, often posing threats to native lizard species (Weterings and Vetter 2018), indicating a high likelihood of establishment and potential ecological impacts in the rapidly urbanizing southern regions of China. The successful invasion history of *I. iguana* in Florida and several Caribbean islands indicates its capacity for rapid population growth and ecosystem impacts when introduced to suitable habitats (Claunch et al. 2025). The species' ability to adapt to both natural and modified landscapes, combined with its popularity in the pet trade, makes it a significant concern for southern China's ecosystem security (Lee et al. 2019; Mo and Mo 2022). This spatial variation in invasion risk highlights the importance of species‐specific assessments in developing targeted management strategies (Early et al. 2016).

Temperature and precipitation are key environmental factors shaping the distribution of invasive ectothermic species, whose physiology and activity are strongly constrained by thermal and hydric conditions (Buckley et al. 2012; Sunday et al. 2011). Although behavioral plasticity (Doody et al. 2019) and selection for cold‐tolerant genotypes (Chang et al. 2022) may enable some species to overcome thermal constraints, winter temperature remains a critical factor limiting their potential spread, particularly in northern regions (Moore et al. 2023; Sunday et al. 2011). This thermal constraint currently serves as a natural barrier against invasion into cooler regions of China (Domrös and Peng 2012). However, this barrier may become less effective under climate change scenarios (Bellard et al. 2013). These findings align with previous research, reinforcing the critical role of climate in determining the geographic distribution of invasive species (X. Li et al. 2016). The strong association between *A. sagrei* distribution and nonforest vegetation cover highlights its unique invasion ecology compared to other studied species. The species' preference for open, edge habitats and disturbed areas aligns well with landscapes dominated by nonforest vegetation, such as urban parks, agricultural areas, and residential gardens (Kolbe et al. 2016; Stroud et al. 2017). The species' ability to thrive in human‐modified landscapes suggests that its invasion potential may be enhanced by anthropogenic landscape transformation, making it particularly challenging to manage in developing regions (Romero et al. 2025).

Our future projections indicate a concerning trend of increasing invasion risk across broader geographical areas for all studied species. The potential expansion of suitable habitats under climate change scenarios suggests that areas currently protected by thermal barriers may become vulnerable to invasion in the future (Bellard et al. 2018; Urban et al. 2016). This is particularly evident in the projected northward and inland shifts of suitable habitats, indicating that regions previously considered low risk may face greater invasion pressure in coming decades. Species showing larger potential range expansions under future scenarios may require more immediate attention in prevention and control strategies (Beaury et al. 2020; Salva and Bradley 2023). These findings emphasize the importance of incorporating climate change projections into long‐term invasion risk assessments and management planning (Hulme 2017; Walther et al. 2009).

The five invasive reptile species assessed in this study pose notable ecological risks to native ecosystems in China. *Anolis sagrei* exhibits strong competitive ability and behavioral dominance, often displacing native lizards and reshaping prey communities in invaded areas (Romero et al. 2025). *Hemidactylus brookii* thrives in urban environments, where artificial lighting and human structures facilitate its dominance and enable competitive exclusion of native geckos, potentially reducing native species abundance and altering nocturnal insect communities (Weterings and Vetter 2018). *Chamaeleo calyptratus* and *I. iguana* are large‐bodied omnivores capable of modifying vegetation structure and nutrient cycling through broad diets and opportunistic predation (Claunch et al. 2025; Dalaba et al. 2019; Falcón et al. 2012). *Gekko monarchus*, although currently limited in range, is strongly associated with human settlements and shows high fecundity and adaptability to artificial habitats, forming competitive interactions with native geckos in urban environments (Lee et al. 2019; Quah et al. 2022). These ecological traits highlight the need for proactive and species‐specific management strategies. To prevent non‐native reptiles, particularly high‐risk species, from further spreading to other regions of China from Hong Kong and Taiwan, we propose the following management recommendations. For small‐bodied urban invaders such as *A. sagrei* and *H. brookii* , priority should be given to strengthening biosecurity and quarantine measures along major transportation routes connecting Hong Kong and Taiwan to mainland China (Agarwal et al. 2021; Alvarez et al. 2024). For large‐bodied species frequently introduced through the pet trade, such as *C. calyptratus* and *I. iguana* , strict regulation of the pet industry and rapid‐response eradication programs are essential to prevent pet‐mediated invasions and the establishment of wild populations in peri‐urban and natural areas (Lockwood et al. 2019; Robinson et al. 2015). For *G. monarchus* , which shows a relatively low invasion risk in our models, maintaining routine monitoring is sufficient. Additionally, establishing an integrated monitoring network across southern Chinese provinces adjacent to Hong Kong and Taiwan will be crucial for coordinated detection and management of potential invasions. This network should facilitate information sharing on species occurrences and joint control actions among local authorities, particularly in regions projected to become increasingly suitable under future climate scenarios (Vicente et al. 2016). Finally, raising public awareness of the ecological risks posed by exotic reptiles—especially among pet owners and traders—and promoting community participation in species reporting will be vital to strengthen long‐term prevention and rapid response (Encarnação et al. 2021).

This study has several limitations. Our models primarily rely on climatic and land cover variables, but other important factors such as biotic interactions, species dispersal abilities, and human‐assisted dispersal were not directly incorporated (Hulme 2009; Travis et al. 2013; Wisz et al. 2013). Because our modeling approach was based on global occurrence data, we did not include anthropogenic variables such as economic activity or urbanization, which mainly influence species distributions in their introduced ranges rather than across their entire global extent (Gallardo et al. 2015). Consequently, our results should be interpreted as indicative of potential habitats delineated solely by abiotic environmental factors. Furthermore, although future climate scenarios were incorporated, uncertainties in climate projections and the potential adaptive responses of species to climate change were not thoroughly captured by our model (Buisson et al. 2010; Hoffmann and Sgrò 2011). Future research directions should address several key aspects. First, incorporating dispersal mechanisms and invasion pathways into distribution models would provide more realistic predictions of spread patterns (Gallien et al. 2010). Second, field validation studies in predicted high‐risk areas would help verify model predictions and assess actual establishment potential (West et al. 2016). Finally, studies examining the competitive interactions between invasive reptiles and native species would also enhance our understanding of potential ecological impacts (Bellard, Cassey, and Blackburn 2016; Doherty et al. 2016).

## 5 Conclusions

This study provides the first comprehensive assessment of invasion potential for five established invasive reptiles from Hong Kong and Taiwan in mainland China. Our findings indicate clear species‐specific differences, with *A. sagrei* exhibiting the greatest potential for widespread invasion. Moreover, climate change may drive northward and inland shifts, likely expanding future invasion risks beyond current geographic limits. These results underscore the urgent need for targeted monitoring, adaptive biosecurity measures, and region‐specific management strategies. Future research should address limitations such as spatial biases and the exclusion of biotic interactions by integrating dispersal mechanisms, conducting field validations, and evaluating competitive dynamics between invasive and native species.

## Author Contributions

**Chaosheng Mu:** conceptualization (lead), data curation (lead), formal analysis (lead), investigation (lead), methodology (lead), software (lead), validation (lead), visualization (lead), writing – original draft (lead). **Jichao Wang:** conceptualization (supporting), funding acquisition (lead), supervision (lead), writing – review and editing (lead).

## Conflicts of Interest

The authors declare no conflicts of interest.

## Data Availability

The species occurrence dataset employed in this investigation was obtained from Mi et al. (2023), accessible at https://doi.org/10.6084/m9.figshare.20958190.v1. Bioclimatic and elevation variables were acquired from the WorldClim database (https://www.worldclim.org/data/index.html). Land cover data were derived from studies by Chen et al. (2022) and Chen et al. (2020), accessible at https://doi.org/10.5281/zenodo.4584775 and https://doi.org/10.25584/data.2020‐04.1190/1615771, respectively.
